# Magnetic resonance imaging characteristics of small cell and non-small cell lung cancer brain metastases: a retrospective study

**DOI:** 10.25122/jml-2024-0411

**Published:** 2025-06

**Authors:** Daniela Pomohaci, Emilia Adriana Marciuc, Bogdan-Ionuț Dobrovăț, Mihaela-Roxana Popescu, Diana-Andreea Ilinca, Costin Chirica, Oriana-Maria Oniciuc (Onicescu), Danisia Haba

**Affiliations:** 1 General and Dental Radiology, Faculty of Medicine, Grigore T Popa University of Medicine and Pharmacy, Iasi, Romania; 2 Radiology Department, Emergency Hospital Prof Dr Nicolae Oblu, Iasi, Romania; 3 Doctoral School, Faculty of Medicine, Grigore T. Popa University of Medicine and Pharmacy, Iași, Romania; 4 Doctoral School, Faculty of Computer Science, Alexandru Ioan Cuza University, Iași, Romania

**Keywords:** magnetic resonance imaging, machine learning, brain metastases imaging, lung cancer, NSCLC, SCLC, ADC, Apparent Diffusion Coefficient, ASC, Adenosquamous Carcinoma, BMs, Brain Metastases, CE T1-W, Contrast-Enhanced T1-Weighted, CSF, Cerebrospinal Fluid, DWI, Diffusion-Weighted Imaging, FLAIR, Fluid-Attenuated Inversion Recovery, FSPGR, Fast Spoiled Gradient-Recalled Echo, HS, Hippocampal Sparing, LC, Lung Cancer, MRI, Magnetic Resonance Imaging, NSCLC, Non-Small Cell Lung Cancer, SCLC, Small Cell Lung Cancer, SRT, Stereotactic Radiotherapy, SWAN, Susceptibility-Weighted Angiography, SWI, Susceptibility-Weighted Imaging, T1-W, T1-Weighted, T2-W, T2-Weighted, WBRT, Whole-Brain Radiotherapy

## Abstract

Brain metastases (BMs) from bronchopulmonary tumors are a major cause of morbidity and mortality and significantly reduce the quality of life in oncology patients. Their treatment depends on imaging features (size, number, location) and their genetic mutation subtype, small-cell lung cancer (SCLC) or non-small cell lung cancer (NSCLC). In patients with SCLC, prophylactic whole-brain radiotherapy (WBRT) with hippocampal sparing (HS) is recommended, whereas in patients with NSCLC, systemic targeted therapy is preferred. Multiple studies have analyzed the MRI morphology of BMs from both SCLC and NSCLC to identify specific imaging characteristics that can guide the selection of appropriate treatment. However, data on lung cancer (LC) brain metastases in patients from Romania are scarce or nonexistent. Our purpose was to investigate the imaging features of both NSCLC and SCLC BMs in our population using conventional MRI protocols. We selected patients from our hospital between 2019 and 2023 who had a histopathological diagnosis of LC BMs and underwent complete MRI exams prior to any radiotherapy or surgical treatment. For every MRI feature, we created both numerical and categorical variables, which were further studied using univariate, bivariate, and multivariate analyses, as well as a machine learning algorithm. We found 62 patients (49 men, 79.03% and 13 women, 20.96%) with confirmed LC BMs, of which 53 (85.49%) had NSCLC and 7 (11.29%) had SCLC. The sites affected were the cerebral hemisphere (56.46%), the cerebellum (40.32%), and the deep nuclei (6.45%), with the latter affecting relatively younger patients (*P* = 0.01), most notably in the case of thalamic situs (*P* = 0.0001). The SCLC subgroup showed a *P* value of 0.025 for the number of lesions, indicating diffuse spread. The AI algorithm identified positive and negative imaging diagnostic prediction variables, including internal vascularization and the number of lesions, respectively, as well as cystic lesions and internal hemorrhage. Further multicentric studies are needed to unravel the MRI features of LC BMs.

## INTRODUCTION

### Lung cancer

Lung cancer (LC) is the most frequently diagnosed primary neoplasm, accounting for approximately 12.4% of all tumors, and is associated with high mortality rates [1,2]. Based on genetic mutations, bronchogenic carcinomas are classified as either non-small cell lung cancer (NSCLC), which represents 62–85% of LC cases, or small cell lung cancer (SCLC), which accounts for 14–39% [2-4]. According to the 2021 World Health Organization (WHO) Classification of Thoracic Tumors, NSCLC subtypes include adenocarcinomas (~50%), squamous cell carcinomas (~30%), and other rare forms [2,5,6]. Molecular profiling of bronchogenic tumors plays a major role in medical decision-making by enabling the selection of suitable systemic target therapies [7].

### Lung cancer brain metastases

Up to 36–64% of brain metastases (BMs) derive from primary bronchogenic carcinoma [8]. The presence of parenchymal BMs in oncologic patients reduces their overall survival to 6–9 months [9,10] and to 3 months in older patients (>65 years old) [7].

Approximately 80% of patients with SCLC will develop BMs in their lifetime, and 20–30% of cases will be diagnosed with synchronous secondary brain lesions, pronounced at the same time as the primary tumor [2,8]. As the risk of secondary brain lesion development is high and SCLC is highly responsive to radiotherapy, prophylactic whole-brain radiotherapy (WBRT) with hippocampal sparing (HS) is recommended for these patients [4,7,9]. In contrast, 30-50% of BMs derive from NSCLC [4]. If the secondary lesion is unique, local management can be accomplished; in cases of multiple lesions, systemic target therapies are administered, depending on the molecular type of NSCLC [7,11]. The early diagnosis of secondary brain lesions and their treatment improves quality of life [12]. Given the distinct clinical approaches required for SCLC and NSCLC patients with parenchymal brain metastases, differentiating between these two tumor types is essential [4,9,13].

### Magnetic resonance imaging

Magnetic resonance imaging (MRI) has a notable role in assessing both anatomical and functional characteristics of brain lesions, including BMs. The most commonly used and essential MRI sequences in BMs diagnosis include T1-weighted (T1-W) imaging with intravenous administration of paramagnetic contrast agent (gadolinium), known as contrast-enhanced T1-weighted (CE T1-W) imaging. It provides information about the anatomical site, volume, number, and amount of gadolinium administered. T2-weighted fluid-attenuated inversion recovery (T2-W FLAIR) imaging is used to evaluate the extent of surrounding vasogenic edema [14]. Advanced MRI sequences like diffusion-weighted imaging (DWI) and apparent diffusion coefficient (ADC) maps are invaluable tools in understanding the cellularity of BMs, by assessing the anisotropic flow of water particles, with lower ADC values in increased cellular density and high ADC values in cellular necrosis [13]. Susceptibility-weighted imaging (SWI/SWAN) can estimate the vascularization or intralesional hemorrhage determined by the 'blooming' of deoxyhemoglobin and iron, paramagnetic substances, and calcifications [15].

BMs on MRI are highly heterogeneous, depending on the type of primary tumor, but they share some common features. Typically, BMs appear edematous, with hypo- or isointense T1-w and T2-w signals, a hypointense T2-w FLAIR signal compared to gray matter, and ring-like or nodular intense enhancement [16]. The intense contrast intake is due to blood-brain barrier rupture and increased blood supply by neo-vascularization [4].

Given the complex capabilities of MRI, it can be used as a diagnostic device to monitor treatment response, tumor recurrence, or progression [4]. Numerous studies have implemented MRI protocols to better characterize lung cancer BMs, focusing on SCLC [4,9], NSCLC [17], or both subtypes [6,13,18].

This study aimed to analyze and describe the LC BMs MRI features (situs, number, volume, cellularity, vascularization, and enhancement) of NSCLC and SCLC, in order to improve diagnostic sensitivity for this type of BMs and inform therapeutic protocol choice. Another objective was to find a correlation between secondary lesions from bronchogenic cancer and their MRI features in order to help clinicians and radiologists differentiate them on a morphological level.

## MATERIAL AND METHODS

### Patient selection

We conducted a retrospective, single-center, observational study from January 2019 to December 2023, assessing the radiological characteristics of secondary brain lesions associated with primary lung cancer. We included adult patients who met the inclusion and exclusion criteria. The inclusion criteria were: (1) age greater than 18 years, (2) histopathological confirmation of BMs from bronchogenic carcinoma, and (3) complete MRI examination prior to surgical treatment or radiotherapy. If the patient had multiple lesions and only one was resected, they were eventually included because of the possibility of analyzing the remaining lesions. The complete investigation consisted of conventional 1.5 T MRI protocols, including T1-W, T2-W, T2-W FLAIR, CE T1-W, SWI/SWAN, and diffusion-weighted imaging with apparent diffusion coefficient mapping (DWI/ADC) sequences. We excluded patients with (1) incomplete MRI examinations that lacked one or more of the previously mentioned sequences; (2) patients who had only one metastasis that was previously surgically treated; and (3) patients who underwent radiotherapy, regardless of the number of secondary lesions.

### MRI acquisitions

The MRI examinations were performed using a Signa Explorer system from General Electric Medical Systems, operating at a field strength of 1.5 Tesla. Acquisition parameters varied depending on the sequence and are detailed in Supplementary Table 1. Although there were patients with both native three-dimensional fast spoiled gradient echo (3D FSPGR) T1-W and gradient echo T1-W (Sag 3D T1), the first sequence was usually preferred after contrast intake because it gives a tridimensional view of the brain and can decrease vascular signal, thereby minimizing the risk of confounding a cross-sectional view of a blood vessel for a small-diameter metastasis [16].

Supplementary tables and figures

### Image analysis

For every patient included in the study, we analyzed the MRI images and manually assessed the number, size (volume), the situs of the lesions, and the presence of peripheric vasogenic edema (hyperintense T2-W and T2-W FLAIR signals). We subdivided the type of contrast enhancement into peripheral or ring-like in cystic lesions, solid with central necrosis, or mixed for cystic lesions with peripheral solid components. We also evaluated the diffusion restriction (DWI/ADC) and the vascularization and/or intralesional bleeding (SWI/SWAN) of the lesions. Each enhancement type was considered to reflect a distinct pattern of lesion evolution. Solid lesions were characterized by an enhancing mass with central necrosis, cystic lesions were characterized by the formation of cystic cavities into the brain, appearing as ring-like enhanced tumors with liquid contents (e.g., blood, cerebrospinal fluid-like material, or proteinaceous fluid), and mixt lesions displayed both cystic and eccentric solid components, with variable proportions of each.

### Statistical analysis

We created a table in Excel that contained both numeric and categorical attributes. We selected 33 features describing the dataset: sex, age at diagnosis, histopathological type of LC, molecular subtype, and the characteristics of the lesions. To statistically investigate the data, we used binary/categorical attributes like molecular subtype of pulmonary cancer (‘NSCLS’ and ‘SCLS’), site (‘supratentorial’, ‘frontal’, ‘parietal’, ‘temporal’, ‘occipital’, ‘infratentorial’, ‘tectum’, ‘midbrain’, ‘pons’, ‘cerebellar’, ‘vermis’, ‘insular’, ‘caudate nucleus’, ‘thalamus’, ‘rostrum’, ‘internal capsule’, ‘external capsule’, ‘intraventricular’), diffusion restriction, type of enhancement (‘solid’, ‘cystic’, ‘mixt’), vascularization and bleeding, and numerical attributes for the age, number of metastases and volume

We then performed univariate analysis, describing each attribute by generating histograms, bivariate analysis to show the relationship between two different binary and numerical attributes, and creating box plots. Additionally, we conducted a multivariate analysis, generating a correlation matrix and calculating the Pearson correlation.

### Univariate analysis

We considered numerical variables and categorical variables. For discrete variables, we computed the percentage of patients in each category. The numerical attributes were age, number, and volume of BMs. Our assumptions were that the number of brain metastases and the volume of the metastasis followed an exponential distribution and that age had a normal distribution [19].

### Bivariate analysis

We examined the relationship between each pair of variables. In order to assess the relationship between binary attributes, we used heatmaps. To identify differences between subgroups defined by binary attributes and continuous variables, we applied statistical tests and generated box plots for data visualization. The number of metastases and lesion volume did not follow a Gaussian distribution; therefore, non-parametric Mann–Whitney U tests were used for these variables. For the age variable, which approximated a normal distribution, a parametric *t*-test was applied. A *P* value of ≤ 0.05 was considered statistically significant [20].

### Multivariate analysis

We generated a 20 × 20 Pearson correlation matrix containing the Pearson correlation coefficient (r) values for each pair of variables. The r value quantifies the strength and direction of the linear relationship between variables as follows: a value of 0 indicates no correlation; values between 0 and ±0.39 represent a weak positive or negative correlation; values between ±0.4 and ±0.69 indicate a moderate correlation; values between ±0.7 and ±0.89 suggest a strong positive or negative correlation; and values between ±0.9 and ±1 show a very strong correlation [21].

### AI learning algorithm

To identify the key variables that contribute to a higher probability of having SCLC BMs, we employed multivariate analysis techniques. We chose to train an Extreme Gradient Boosting (XGBoost) model to predict the presence or absence of SCLC. XGBoost, as an ensemble learning algorithm, is particularly well-suited for imbalanced datasets, as it can handle classes with significantly different frequencies and provide accurate predictions despite the imbalance. Unlike traditional logistic regression or SVMs (Support Vector Machine – a supervised machine learning algorithm), XGBoost dynamically adjusts weights during the training process, enabling it to learn from imbalanced data effectively [22].

### Processing environment

Python (version 3.10.9) was utilized to perform the statistical analysis. The Python packages used were: 'pandas', ‘scipy’, ‘seaborn’, and 'matplotlib'. Demographic differences between the two subgroups were tested using either Student's *t*-test or the Mann–Whitney U test. To measure the linear relationship between the variables, we used the Pearson correlation coefficient (*r*).

## RESULTS

### Primary outcomes

**Figure 1:**
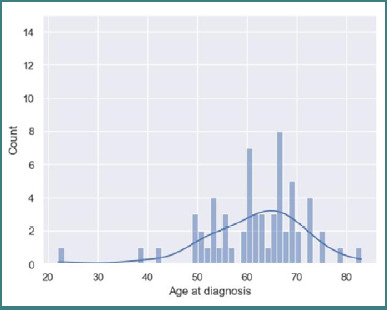
Age at diagnosis histogram

A total of 577 patients were diagnosed with brain metastases between January 2019 and December 2023. Of these, 177 patients had histopathological confirmation of the diagnosis following biopsy or surgical treatment, and 110 patients underwent a comprehensive MRI examination as previously described. Among them, 62 patients were diagnosed with BMs from primary bronchopulmonary cancer, of whom 49 (79.03%) were men and 13 (20.96%) women, with an average age of 61.67 ± 10. 05 years old, including patients from 22 to 83 years old (Figure 1).

**Figure 2:**
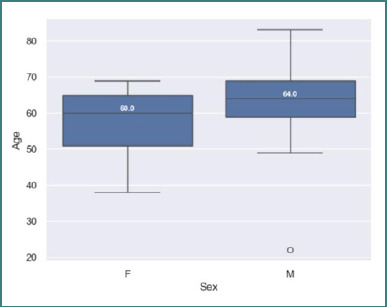
Age of diagnosis in men and women

We identified a statistically significant difference in age between men and women, as determined by the Mann–Whitney U test (*P* = 0.04). The men in this specific population developed secondary brain lesions at a later age, compared to women (60 ± 9.88 SD for women and 64 ± 9.79 SD for men) (Figure 2).

**Figure 3:**
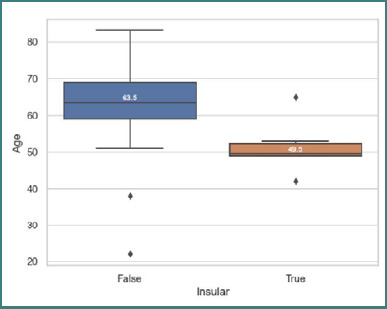
Age at diagnosis of patients with insular location

We also found another statistically significant quantitative difference, with a *P* value of 0.01 (*t*-test) for the insular and deep grey matter situs of tumors, which was more frequent in relatively younger patients, at an age of 49.5 ± 7.6 SD for the insular region and 63.5 ± 9.68 SD for the other situses (Figure 3).

**Figure 4:**
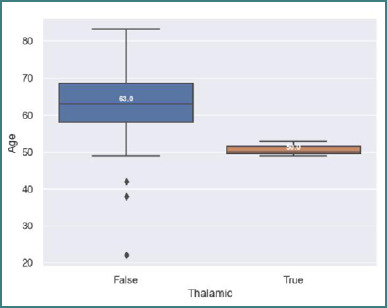
Age at diagnosis of patients with thalamic site

Thalamic secondary lesions were significantly more frequent in younger patients, with a *P* value of 0.0001 (*t*-test). The mean age of patients with thalamic involvement was 50 ± 2.08 years, compared to 63 ± 9.97 years for those with metastases in other brain regions (Figure 4).

**Figure 5:**
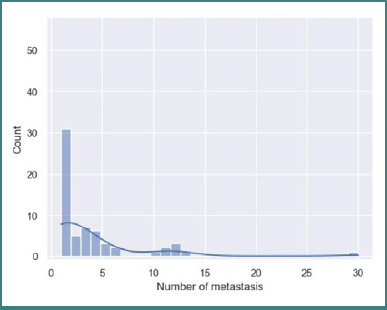
Number of metastases histogram

Regarding the total number of lesions, we observed a considerable range in the numerical values, with an average of 3.61 ± 4.77 standard deviation (SD). The relatively high SD compared to the mean was primarily due to an outlier case with 30 metastases in a single patient, which skewed the mean to the right (Figure 5). We also calculated the median number of lesions, which was 1. Half of the patients (50%, *n* = 31) presented with a solitary metastasis, while the other 50% had multiple brain lesions.

**Figure 6:**
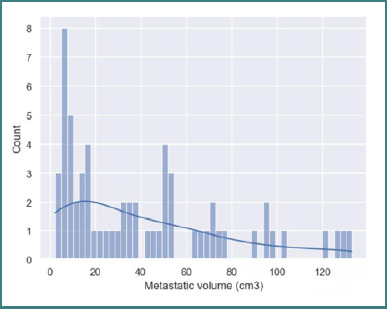
Metastatic volume histogram

The average volume was 41.19 ± 36.03×10^-6^m^3^, and 95% of the population had lesions with a volume between 0 cm^3^ and 41.19+2*36.03×10^-6^m^3^. This indicated that most of the patients (*n* = 59, 95.16%) had multiple smaller lesions and fewer had bigger ones, varying from a volume of 2.39 to 103.89 × 10^-6^m^3^ (Figure 6).

An overview of lesion location showed that 35 patients (56.45%) had supratentorial BMs, 16 (25.81%) had infratentorial lesions, and 11 patients (17.74%) presented with both supratentorial and infratentorial involvement. The supratentorial situses specifically affected were the frontal lobe (34, 54.83%), parietal lobe (13, 20.96%), occipital lobe (9, 14.51%), temporal lobe (17, 27.41%) and the insular region (6, 9.76%), including three patients (4.83%) with thalamic lesions. For the infratentorial locations, the cerebellum was the most affected (*n* = 25 patients, 40.32%), with two cases in the vermis, followed by the midbrain (*n* = 2, 3.22%) and the pons (*n* = 1, 1.61%).

The majority of BMs were from NSCLC, accounting for 53 cases (~85.49%), and seven cases were associated with SCLC (~11.29%). In two cases (3.22%), the primary lung cancer subtype remained unidentified. There was no statistically significant difference in the age at diagnosis between the NSCLC and SCLC subgroups, with a mean age of 63 years in the NSCLC group and 60 years in the SCLC group.

### NSCLS

In the NSCLC subpopulation, 42 were men (67.74%) and 11 were women (17.74%). The greater part of NSCLC secondary lesions was supratentorial (39, 62.90%), of which 28 patients had lesions in the frontal lobe (45.16%), 15 in the temporal lobe (24.19%), 11 in the parietal lobe (17.74%), seven in the occipital lobe (11.29%), and one subependymal lesion in the right lateral ventricle (1.61%) from a squamous cell carcinoma. Three patients had BMs in the deep grey matter, one in the rostrum (1.61%) and two in the thalamus (3.22%). Twenty-three patients had infratentorial BMs (37.09%), with 21 located in the cerebellum (33.87%), of which one was in the vermis, and only two in the midbrain (3.22%).

**Figure 7:**
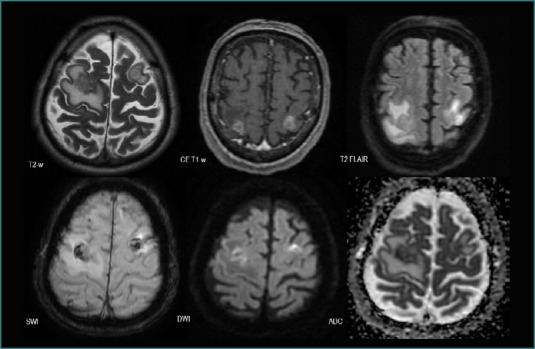
MRI brain acquisition of an NSCLC patient (adenocarcinoma)

Regarding their features, NSCLC metastases tended to be solid lesions with central necrosis (*n* = 52, 83.87%), associated with peripheral edema (*n* = 52, 82.87%), and showed diffusion restriction (*n* = 34, 54.83%). They also developed neovascularization (*n* = 34, 54.83%) or had intralesional bleeding (11.29%) (Figure 7).

The MRI shows two edematous solid lesions in the frontal lobes (high T2-w and T2 FLAIR signals in the surrounding white matter), with intralesional bleeding (‘blooming’ on SWI), predominantly peripheric contrast intake (CE T1-w), and diffusion restriction (high DWI and low ADC) suggesting central necrosis.

### SCLC

In the SCLC subpopulation, five patients were men (8.06%) and two were women (3.22%). Four patients had secondary tumors located in the supratentorial region, two had exclusively infratentorial lesions, and one patient presented with both supratentorial and infratentorial metastases. The supratentorial sites included the frontal lobe (four patients, 6.45%) and the parietal, occipital, and temporal lobes (two patients each, 3.22%). Infratentorial sites included the cerebellum (four patients, 6.45%) and the pons (1 patient, 1.61%). Only two patients had BMs in the insula (3.22%), one of whom also had a lesion in the thalamus.

**Figure 8:**
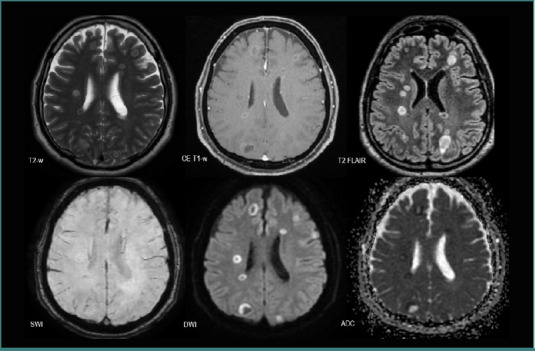
MRI brain acquisition of BMs from an SCLC patient. Multiple small lesions bilaterally scattered, with low peripheric edema (discrete high T2-w FLAIR signal), no ‘blooming’ on SWI, intense peripheric diffusion restriction (high DWI and low ADC), and mild contrast intake (CE T1-w).

All the patients with BMs from SCLC displayed lesions with poor intralesional neovascularization (*n* = 7, 11.29%), six patients had mixed components and were poorly edematous (9.67%), four of them had diffusion restriction (6.45%), and generally no intralesional hemorrhage (Figure 8). Only one patient displayed intralesional hemorrhage.

### NSCLC vs SCLC

Regarding the total number of secondary lesions, there was a statistically significant difference (*P* = 0.025) between the two groups, SCLC displaying a diffuse spreading with numerous BMs, as seen in Figure 8. In contrast, no significant difference was observed in terms of lesion volume (*P* = 0.10, Mann–Whitney U test), with an average volume of 29.53 × 10^-6^ m^3^ for the NSCLC group and 35.02 × 10^-6^ m^3^ for the SCLC group.

The Pearson correlation matrix revealed a weak negative relationship between the insular situs and patient age (*r* = -0.34) and a weak positive relationship between the number of lesions and the SCLC subtype of LC (*r* = 0.35). It also showed the prevalence of mixt lesions in male patients (*r* = 0.35), a moderate positive relation between occipital situs and both insular situs (*r* = 0.48) and temporal situs (*r* = 0.47); and a weak one with the number of BMs (*r* = 0.37); a weak positive correlation between frontal situs and high cellularity (*r* = 0.34). The SCLC subgroup showed a weak positive correlation with the insular location (*r* = 0.23) and with the presence of neovascularization (*r* = 0.25) (see Supplementary Figure 1). We also observed an *r* value of -0.70 between ‘intraventricular’ and ‘edematous’, indicating that if a lesion develops subependymally, it is very unlikely to result in edema.

### AI implementation

**Figure 9:**
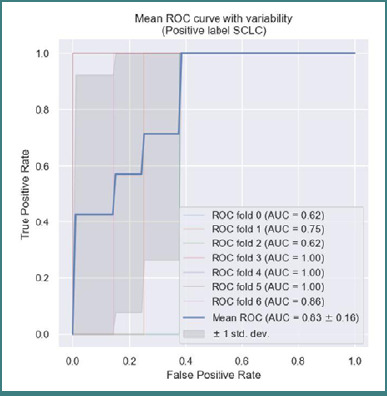
ROC curve of XGBoost accuracy to identify SCLC BMs

The XGBoost model achieved a high AUC-ROC score of 0.83 (95% CI, 0.826–0.853), evaluated using cross-validation. This indicates a 'very good' predictive performance [23], with an accuracy ranging between 0.8 and 0.9, a specificity of 0.94, and a sensitivity of 1 (Figure 9). These results suggest that the model is well-suited for identifying BMs originating from SCLC, with key predictive variables including cystic lesion morphology, number of metastases, and vascularization. The score of feature importance derived from the XGBoost model is shown in Supplementary Figure 2. As shown in this figure, location, peripheral edema, diffusion restriction, and sex had no predictive value. The variables with the highest predictive value were the histological subtypes; however, these have no diagnostic utility in this context, as they were predefined at the outset, but they confirm XGBoost's capacity to identify predictive values

**Figure 10:**
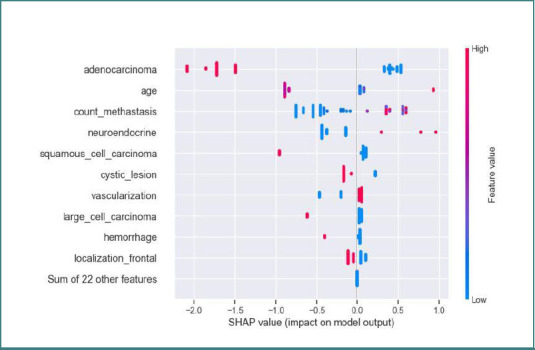
SHAP plot showing the impact of features on the predictive model

SHAP analysis (Shapley Additive exPlanations) was used to identify the most influential variables in predicting SCLC presence in the trained XGBoost model [24]. The impact of the variables and their negative or positive predictive nature can be seen in Figure 10. A positive predictive influence was associated with a high number of lesions and the presence of internal neovascularization, whereas a negative predictive influence was linked to the predominance of cystic lesions and internal hemorrhage. Other variables had little or no predictive value.

### Secondary outcomes

According to the 2021 WHO Classification of Lung Tumors, histopathological subtypes of NSCLC in our cohort were adenocarcinoma (*n* = 30, 48.38%), squamous cell carcinoma (*n* = 9, 14.51%), large cell carcinoma (*n* = 8, 12.90%), sarcomatoid subtype (*n* = 2, 3.22%), neuroendocrine (*n* = 1, 1.61%), one adenosquamous carcinoma (ASC) (1.61%) and two unknown (*n* = 2, 3.22%) [5].

### Adenocarcinomas

**Figure 11:**
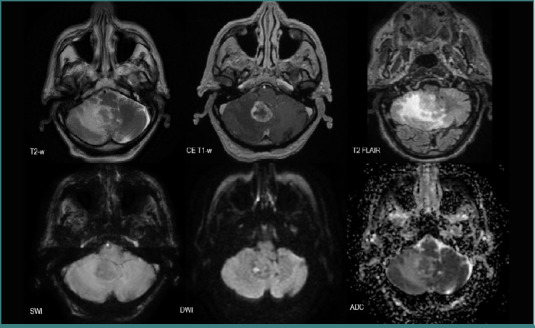
MRI brain acquisition of an NSCLC patient (adenocarcinoma). Highly edematous solid cerebellar lesion (high T2-w and T2-w FLAIR signals in the surrounding white matter) compressing the fourth ventricle. Discreet ‘blooming’ on SWI and diffusion restriction limited to a small area of the tumor (high DWI and low ADC). Peripheral contrast enhancement on the CE T1-W image suggests central necrosis.

50% of supratentorial BMs originated from adenocarcinomas (23 patients, 37.09%) and were situated mainly in the frontal lobe (17 patients, 27.41%). Infratentorial adenocarcinoma BMs were mainly found in the cerebellum (11 patients,17.74%), and most NSCLC BMs in the deep gray nuclei were from adenocarcinomas. BMs were predominantly solid with central necrosis, exhibiting neovascularization but without intralesional bleeding (found in only three lesions), and showed diffusion restriction and peripheral edema (Figure 11).

### Squamous cell carcinoma

According to our findings, squamous cell carcinoma may tend to disseminate into the brain parenchyma in older patients, with a *P* value of 0.054 from the *t*-test, which was not statistically significant.

**Figure 12:**
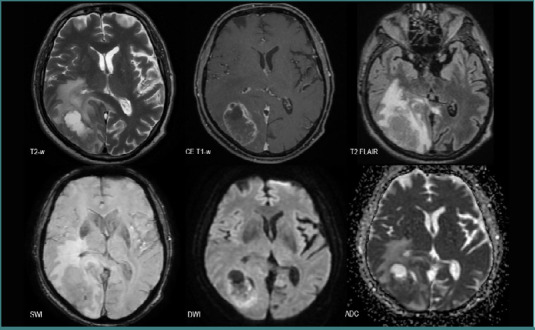
MRI brain acquisition of a patient with squamous cell carcinoma. Voluminous right parieto-occipital lesion, with important central necrosis and cystic component (the area with high T2-w, low CE T1-w signals and facilitated diffusion – low DWI and high ACD signals). It also displays poor intralesional vascularization (a small spot on SWI), significant peripheral edema (high T2-w and T2-w FLAIR signals), and diffusion restriction of the remaining solid component (high DWI and low ADC).

BMs from squamous cell carcinoma were mainly edematous and mixed lesions with a cystic component (8/9 patients, 12.90%). They demonstrated intralesional vascularization in six patients (9.67%), and none of them exhibited intralesional bleeding on SWI (Figure 12).

**Figure 13:**
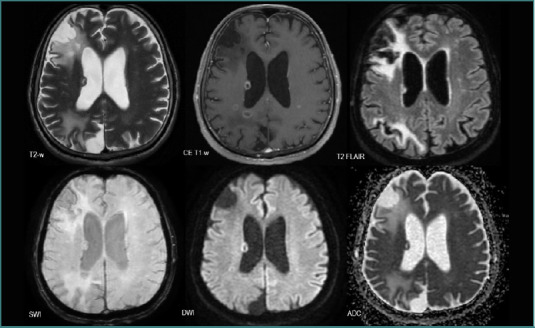
MRI brain acquisition of a patient with squamous cell carcinoma. Multiple lesions and a subependymal one on the external wall of the right lateral ventricle, with intense peripheric diffusion (high DWI and low ADC) and contrast intake with central necrosis (CE T1-w), without intralesional vascularization (SWI) and surrounding edema (T2-w, T2-w Flair). To note two porencephalic areas with peripheric gliosis suggesting ischemic stroke sequelae (right frontal and parietal).

They were located equally in both supratentorial and infratentorial regions. There was one particular case with an intraventricular subependymal squamous cell carcinoma BM (Figure 13).

### Large cell carcinoma

**Figure 14:**
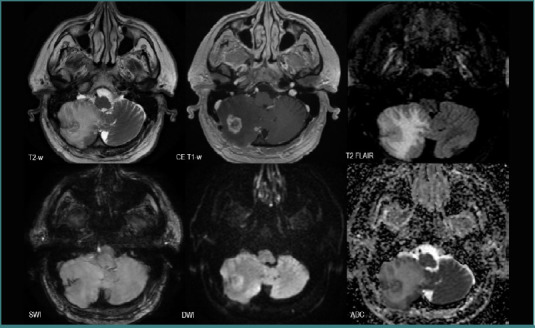
MRI brain acquisition of large cell carcinoma BMs. Highly edematous right cerebellar lesion (high T2-w and T2-w FLAIR signals of the surrounding white matter), with intralesional vascularization (SWI), central necrosis, mild peripheric diffusion (high DWI and low ADC), and contrast intake (CE T1-w).

Large cell carcinoma BMs were prevalent at the supratentorial level, characterized by solid and cystic components, intralesional vascularization, and diffusion restriction, with a high degree of edema (Figure 14).

## DISCUSSION

Lung cancer is the primary tumor responsible for the majority of brain metastases [1,2,4,14,25]. Although there is an overall increase in lung cancer incidence among women, our cohort remains predominantly male [2]. Our findings are consistent with the demographics of LC subtypes, with the majority of secondary brain lesions from NSCLC (85.49%) and only 11.29% from SCLC [1,4,6,7].

Our findings confirmed the metastatic profile of hematogenous spreading, predominantly located in the cerebral hemispheres (56.45%), cerebellar (40.32%), and deep grey matter (6.45%) [10,12].

The frontal lobe was the most frequently affected, as in other findings [9]. NSCLC secondary lesions were prevalently supratentorial (61.9%), specifically frontal and temporal, cerebellar (33.3%), and only 6.3% the insular region [17]. Other studies have confirmed the presence of NSCLC BMs specifically in the cerebellum and frontal lobe, as well as in the parieto-occipital region [9].

The most frequent NSCLC subtypes were adenocarcinoma, large-cell carcinoma, and squamous cell carcinoma, confirming their high tendency to develop brain metastases [6,17], with squamous cell carcinoma displaying a prevalent cystic component.

Although adenocarcinomas involve multiple locations, secondary lesions from SCLC were relatively more numerous, with a diffuse spread, findings consistent with other studies [4,9,17]. These findings are statistically relevant, and they may have resulted from the presence of more than 30 lesions in a single patient. The other six patients had an average of 4.66 lesions, with one patient having a single BM.

The frontal lobe and cerebellum were the primary sites of SCLC dissemination, with the cerebellum and deep structures, especially periventricular localization, being considered high-risk regions [4,9]. A retrospective study by Wang *et al*. also concluded that secondary brain lesions from SCLC have a diffuse distribution, carry the worst prognosis, and that the cerebellum is a high-risk site for developing BMs [9]. These findings may guide the selection of appropriate locations and doses for stereotactic radiotherapy (SRT).

We observed that BMs from LC exhibit their characteristic radiological features more clearly as their volume increases. Initially, these lesions are small in diameter (<5 mm) and can be detected by the presence of perilesional edema (if highly edematous) and through contrast-enhanced (CE) T1-W sequences, where small BMs show strong and homogeneous enhancement. Similar findings were reported in a study focused on SCLC BMs [4]. Zhu *et al*. [4] found that SCLC BMs tend to be more numerous and smaller in diameter compared to NSCLC lesions, but they also displayed significant diffusion restriction, which correlates with a poor prognosis.

As secondary lesions grow beyond approximately 5 mm in diameter, they can develop distinctive features, including cystic components, solid tumors with central necrosis, and intralesional neovascularization. Zhu *et al*. also suggested that increases in lesion volume may be due to intratumoral bleeding, which can be detected using SWI sequences [4].

In addition to size and time of development, the growth rate and metabolic activity of BMs can alter their appearance and the type of enhancement [18]. We identified that BMs from SCLC tend to evolve more rapidly and exhibit central necrosis at a smaller diameter compared to BMs from NSCLC. This finding is not consistent with other studies [4,9], as SCLC BMs can develop internal necrosis relatively late in their development.

Kiyose *el al*. found two growth patterns of LC BMs, one with a higher proliferative status with greater volume, higher cellular density (higher DWI and lower ADC) with low vascularization and low peritumoral edema, consistent with SCLC secondary lesions and one with a lower proliferative status, corresponding to NSCLC metastases [18].

Two other studies confirmed that the primary method for differentiating between the two subgroups is by measuring ADC values, which are significantly lower in SCLC brain metastases [4,13]. Another study concluded that T2-W FLAIR could also be singularly used to differentiate the two groups [6].

In terms of restricted diffusion, our study provides little information, as the analysis was qualitative rather than quantitative; consequently, the XGBoost implementation did not find diagnostic value in DWI/ADC sequences. Our study confirmed some of the SCLC BM features, with most lesions showing internal vascularization, poor peripheral edema, and diffuse spread. However, only internal vascularization and diffuse spread had diagnostic value according to XGBoost.

A unique case of an intraventricular secondary lesion from squamous cell carcinoma of the lung (NSCLC) was discovered (Figure 14). Intraventricular tumor spread is rare, occurring in 1–5% of patients, with a median survival of 1-4 months [7,26]. It could occur by hematogenous dissemination from the extracranial primary tumor or through cerebrospinal fluid (CSF) from another existing BM [27]. They derive predominantly from renal cell carcinomas, breast ductal carcinomas, NSCLC, melanoma, thyroid carcinoma, and colon adenocarcinoma [26,27]. Their management is difficult, as CSF acts both as a barrier for systemic therapies and as a nutrient environment for the metastases. Local surgical treatment is indicated in obstructive hydrocephalus, and radiotherapy is considered in order to minimize leptomeningeal disease [27,28]. In our case, the patients underwent stereotactic radiotherapy, having a small subependymal metastasis on the external wall of the right lateral ventricle, and multiple other parenchymal lesions. NSCLC is not the only pulmonary tumor to disseminate intraventricularly; SCLC can also show this behavior in even more singular cases [29].

A *P* value of 0.054 indicated a tendency for squamous cell carcinoma to spread to the brain in older patients, although it did not reach significance (*P* ≤ 0.05). Further studies focusing on this histopathological subtype may clarify this observation.

**Figure 15:**
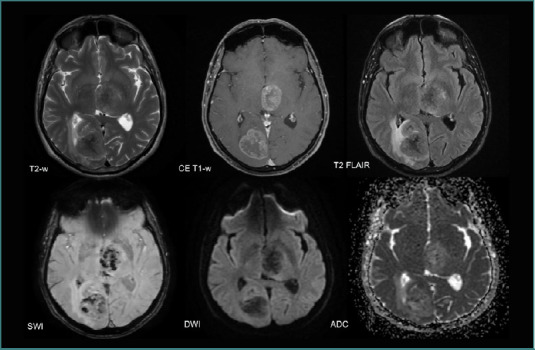
MRI brain acquisition of adenosquamous LC BMs

Among our patients, we identified a rare case of adenosquamous lung carcinoma (ASC) with multiple secondary brain lesions (13 in total), located supratentorially, specifically in the cerebral hemispheres, thalamus, and external capsule/rostrum of the insula with intraventricular extension. The BMs were slightly edematous, solid, and inhomogeneous lesions with rich intralesional vascularization and micro-hemorrhages, with facilitated diffusion and a maximal volume of 51.26 x10^-6^ m^3^ (Figure 15).

Adenosquamous carcinoma (ASC) is a rare subtype of NSCLC, representing only 0.4–4% of LC [30], associated with poor prognosis, with high recurrence of BMs; thus, adjuvant prophylactic WBRT with HS is indicated [2]. The first research to investigate the metastatic spread of ASC is a retrospective study that analyzed more than 800 patients with secondary lesions from this particular histopathological subtype of LC [30]. They found that more than half of the patients had bone metastases, with other common sites including the lung, liver, and brain. Among these, brain metastases were associated with the worst prognosis, which improved only with chemotherapy. Another study that evaluated ASC behavior found that the brain was the primary site for metastatic spread [31]. There is also a case report of gastric metastasis from ASC in a 61-year-old woman [32]. Given these findings, further studies are necessary to gain a deeper understanding of the nature and metastatic behavior of ASC.

### Strengths and limitations

Our study has limitations due to its single-center, retrospective design. We lack a national health registry to track these patients comprehensively, and we do not have complete outcome data for all participants. Additionally, we cannot confirm if patients underwent other diagnostic or treatment procedures unless this information was communicated to their physicians. The fact that we only included histopathologically confirmed BMs from LC was an important step to ensure the quality of our data and also to minimize the potential bias arising from misclassification. We also included complete MRI acquisitions, unlike other studies that analyzed only selected sequences [4,6,9], which makes our results more comprehensive in characterizing the nature of secondary lesions. These steps carry the risk of excluding possible cases, thereby diminishing the size of our cohort and potentially lowering the incidence rates of this illness. The bias arising from the small cohort size was corrected through multivariate analysis and examination of the relationship between model features.

The XGBoost model identified several key variables that have a significant impact on predicting SCLC primary tumors. Given the imbalanced nature of our dataset, with a large proportion of patients with NSCLC, traditional statistical methods would have been insufficient to accurately model this complex relationship.

The retrospective nature of the study is another limitation, as the MRI acquisition parameters differ. It is also important to emphasize that the imaging, reading, and interpretation were manual, not automatically realized, which could also significantly increase the subjectivity of the study.

Because our study was conducted in a single center, it is essential to note that our results should not be generalized, as they do not represent the dynamics of LC BMs in the Romanian population. A higher number of cases is needed to further investigate the morphology of LC BMs subgroups using conventional and functional MRI features, preferably in a multi-center setting.

## CONCLUSION

Our study examined the nature of secondary brain lesions resulting from bronchopulmonary tumors in our population, describing their location, primary features, and identifying their imaging patterns based on their histopathologic characteristics. Our findings suggest that, independent of the histopathological nature of the lung metastases, the absence of or limited perilesional edema, and the early development of central necrosis are associated with a poor prognosis.

The results of our multivariate analysis have important implications for understanding the mechanisms underlying the development of SCLC and NSCLC brain metastases. Further studies are needed for a more comprehensive characterization of SCLC and NSCLC subtypes, using MRI characterization. MRI remains the primary tool for diagnosing secondary brain lesions and other parenchymatous lesions. Its importance in the diagnosis and management of metastatic disease has been previously established.

However, the quantitative and qualitative analysis of conventional MRI features alone cannot accurately identify the subgroup of primary LC, with biopsy remaining the primary diagnostic method. Radiomics and deep learning programs could be helpful in this direction, as suggested by multiple studies in this domain.

## Data Availability

Data from our study is not publicly available. Further data is available from the corresponding author upon reasonable request.

## Personal thanks

We would like to thank the team of MRI technicians who performed the brain MRI acquisitions.
