# Supplementary material for: Magnetic resonance imaging characteristics of small cell and non-small cell lung cancer brain metastases: a retrospective study
Source: J Med Life. 2025 Jun;18(6):563–74. doi: 10.25122/jml-2024-0411 (PMC12314843; doi:10.25122/jml-2024-0411)
Supplement: Supplementary file 1 [file JMedLife-18-563-s001.pdf]

Supplementary tables and figures

Supplementary Table 1. MRI acquisition parameters

Sequences of 1.5 T GE Signa Explorer	Slice thickness (mm)	Spacing (mm)	TE * (time to echo)	TR * (repetition time)	Echo number	Matrix (rows x columns)
Ax T1 FSPGR 3D	1.8	0.9	4.2	9.72 – 9.88	1	512/512
Sag 3D T1	2	0.9 -1	3.644 -3.676	8.484 – 8.62	1	512/512
Ax T2 Propeller	4	6	124.65	6562.025	1	512/512
	4	5	105.8	7186.8	1	512/512
Sag CUBE 3D Flair	2	1	119 – 119.647	6502	1	512/512
Ax T2 Flair	4	5	123.448	9000	1	512/512
Ax Diffusion b1000	4	5 - 6	85.9 - 86.7	5513 - 6782	1	256/256
ADC	4	-	85.9 – 86.7	5513 - 6782	1	256/256
3D Ax SWAN (SWI)	3	1.5	49.44 -49.776	79.2 – 79.9	1	512/512
Ax T1 FSPGR 3D + C	1.5 – 1.8	0.9	4.2	9.804 – 9.88	1	512/512

* measured in milliseconds

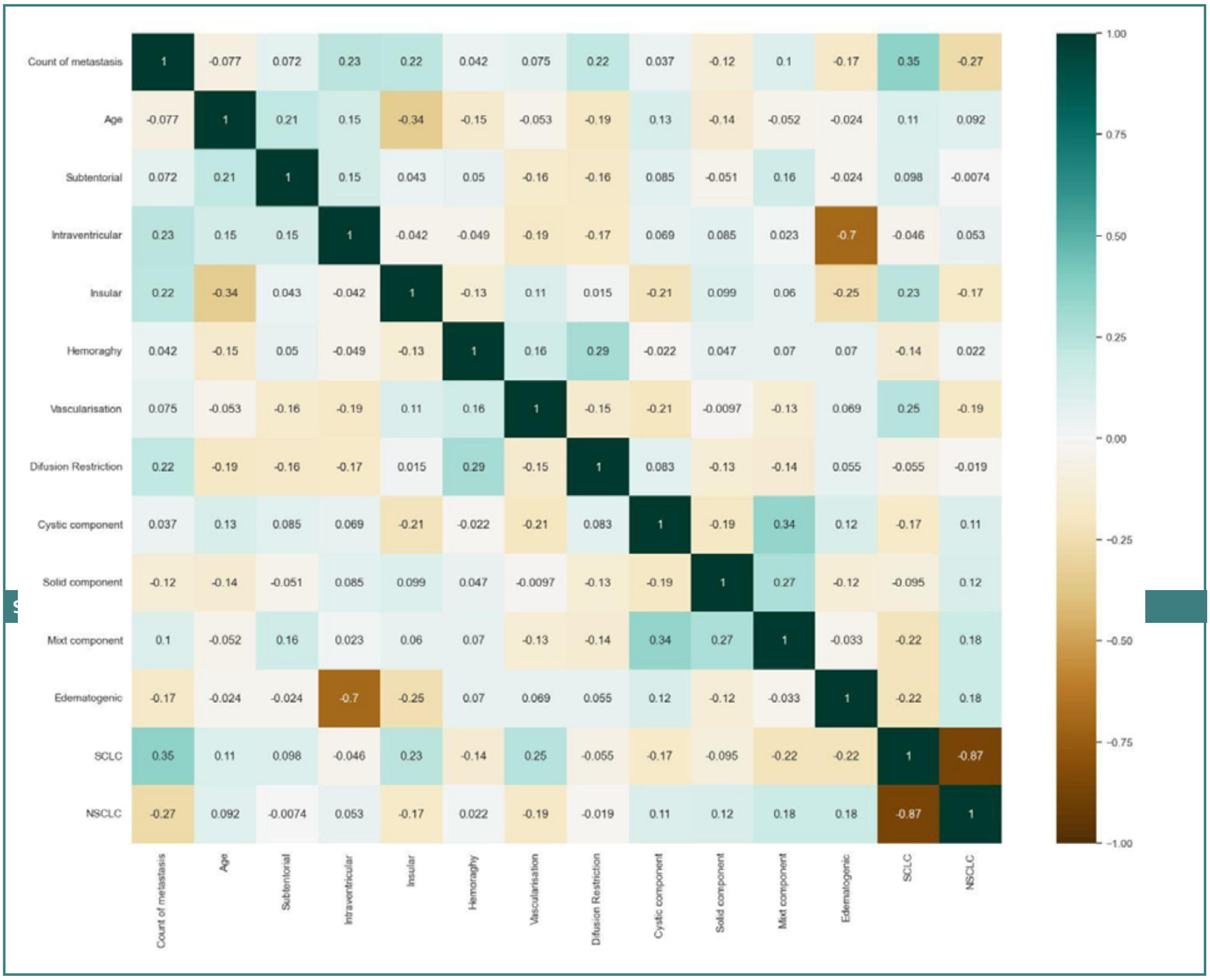

© 2025 by the authors. This article is an open access article distributed under the terms and conditions of the Creative Commons Attribution (CC BY 4.0) license.

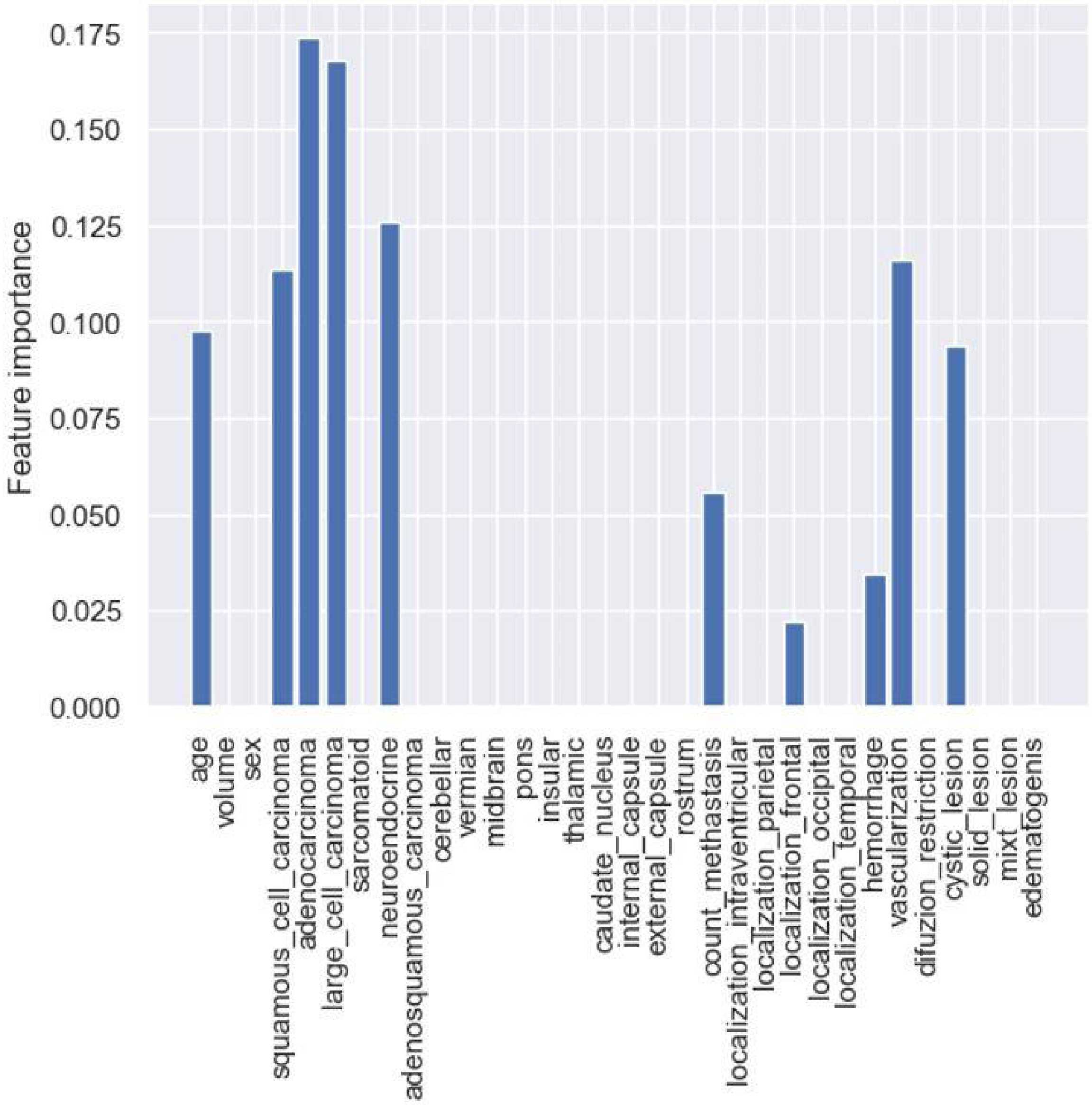

Supplementary Figure 2. Diagram showing relevant features in the prediction model

© 2025 by the authors. This article is an open access article distributed under the terms and conditions of the Creative Commons Attribution (CC BY 4.0) license.
